# Predicting In-Hospital Maternal Mortality in Senegal and Mali

**DOI:** 10.1371/journal.pone.0064157

**Published:** 2013-05-30

**Authors:** Cheikh Ndour, Simplice Dossou Gbété, Noelle Bru, Michal Abrahamowicz, Arnaud Fauconnier, Mamadou Traoré, Aliou Diop, Pierre Fournier, Alexandre Dumont

**Affiliations:** 1 Laboratoire d'Etudes et de Recherches en Statistiques et Développement, Université Gaston Berger, Saint-Louis, Sénégal; 2 Laboratoire de Mathématiques et de leurs Applications – Pau, UMR CNRS 5142, Université de Pau et des Pays de l'Adour, Pau, France; 3 Department of Epidemiology and Biostatistics, McGill University, Montreal, Canada; 4 EA 7285 “Risk and Safety in Clinical Medecine for Women and Perinatal Health”, University of Versailles St-Quentin (UVSQ), Versailles, France; 5 URFOSAME, Referral Health Center of Commune V, Bamako, Mali; 6 International Health Unit, Centre de Recherche du Centre Hospitalier de l'Université de Montréal, Université de Montréal, Montréal, Canada; 7 Institut de Recherche pour le Développement, UMR 216 Mère et enfant face aux infections tropicales, Paris, France; Tehran University of Medical Sciences, Islamic Republic of Iran

## Abstract

**Objective:**

We sought to identify predictors of in-hospital maternal mortality among women attending referral hospitals in Mali and Senegal.

**Methods:**

We conducted a cross-sectional epidemiological survey using data from a cluster randomized controlled trial (QUARITE trial) in 46 referral hospitals in Mali and Senegal, during the pre-intervention period of the trial (from October 1st 2007 to October 1st 2008). We included 89,518 women who delivered in the 46 hospitals during this period. Data were collected on women's characteristics, obstetric complications, and vital status until the hospital discharge. We developed a tree-like classification rule (classification rule) to identify patient subgroups at high risk of maternal in-hospital mortality.

**Results:**

Our analyses confirm that patients with uterine rupture, hemorrhage or prolonged/obstructed labor, and those who have an emergency ante-partum cesarean delivery have an increased risk of in-hospital mortality, especially if they are referred from another health facility. Twenty relevant patterns, based on fourteen predictors variables, are used to predict in-hospital maternal mortality with 81.41% sensitivity (95% CI = [77.12%–87.70%]) and 81.6% specificity (95% CI = [81.16%–82.02%]).

**Conclusion:**

The proposed class association rule method will help health care professionals in referral hospitals in Mali and Senegal to identify mothers at high risk of in-hospital death, and can provide scientific evidence on which to base their decisions to manage patients delivering in their health facilities.

## Introduction

In Sub-Saharan Africa (SSA), maternal mortality and morbidity are major problems for which progress has been inadequate [1]. The broad strategies that have made it possible to reduce maternal mortality are known: prenatal care, labor and delivery management by qualified birth attendant, and availability of emergency obstetric care (EmOC) [2]. However, their implementation is a major challenge in Sub-Saharan Africa, where health care systems are fragile and still being developed, so that service availability and quality of care in health facilities are very heterogeneous and most often inadequate [3]. The qualification of health personnel working in EmOC services is highly variable, yet it has a major impact on maternal and prenatal outcomes: more the staff is qualified, better is the survival of both the mothers and the newborns [4].

Hospital-based maternal mortality in SSA countries varies markedly between studies (0.1–5%) [5], [6], [7], [8], [9], [10], [11], depending upon the type of hospital (regional or district hospital), the setting (rural or urban), and the characteristics of the women (case mix). Hemorrhage, hypertensive disorders, protracted labor including uterine rupture, puerperal infection and abortion complications are the main cause of hospital-based maternal deaths. The majority of deaths occur in the first two days after the birth [12]. While there are numerous factors that contribute to maternal mortality, most of researchers have focused on those that affect the interval between the onset of obstetric complication and its outcome. These factors are grouped in three categories [13]: factors that affect (1) delay the decision to seek care; (2) delay arrival at a health facility; and (3) delay the provision of adequate care.

However, different studies from SSA countries identified many independent risk factors which differed substantially between authors, probably due to differences in patient populations, setting, variables collected and statistical methods. Thus, it remains difficult to provide health professionals from SSA countries with recommendations to identify clinical signs or symptoms which could help the staff to detect patients with high risk of in-hospital death. Yet, such criteria will help staff to decide whether a patient should be managed as a high priority case by qualified health professionals in comprehensive EmOC [2]. Although the criteria of severe obstetric complications are proposed by the World Health Organization (WHO) as appropriate predictors of maternal mortality [14], difficulties remain in their identification, and there is limited experience with the use of these criteria in low-income countries [15].

The QUARITE trial is an international multi-center cluster randomized trial designed to assess the efficacy of a quality care improvement program in Senegal and Mali, compared with a control ‘usual-care’ group without external intervention [16]. The primary endpoint of the trial is maternal in-hospital mortality. With about 80,000 births occurring annually in 46 referral hospitals, QUARITE is one of the largest cluster randomized trial in maternal and perinatal health ever to be undertaken in low-income countries. Thus, the trial provides an unique opportunity to evaluate in-hospital maternal mortality from a large number of centers, in a variety of contexts and taking into account different maternal and hospital characteristics. In this article, we aimed to measure maternal mortality in referral hospitals in Mali and Senegal before the implementation of the quality care improvement program and to assess predictors of in-hospital mortality in patients attending these health facilities.

## Methods

### Study design and population

We conducted a cross-sectional epidemiological survey using data collected during the pre-intervention phase of a cluster-randomized controlled trial (QUARITE trial) in 46 referral hospitals (22 in Mali and 24 in Senegal). The study setting and methods were published in detail at the trial's inception [16]. The trial aims to assess the effectiveness of the ALARM international program (AIP) – Which is a multifaceted intervention based on maternal death reviews – in reducing in-maternal mortality. All procedures were approved by the ethics committee of Sainte-Justine Hospital in Montreal, Canada and by the national ethics committees of Mali and Senegal. However data are not made yet available to the scientific community, because all the analyses of the QUARITE trial are not finished. First-level and second-level public referral hospitals with more than 800 deliveries per year that had a functional operating room were eligible to participate. Centers were included on the basis of formal, informed consent on the part of the hospital director and the person in charge of maternity services. All women who delivered in each of the participating facilities from October 1, 2007 to September 30, 2008 in Senegal and from November 1, 2007 to October 31, 2008 in Mali were included. Women who delivered at home or in another centre with postnatal transfer were excluded. Patients were followed up in the hospital until their discharge.

Since this study was carried out during the pre-intervention phase of the trial, there were no specific recommendations from the researchers to the health personnel. It was expected that labor management, diagnosis and treatment of complications were based on national or international standards. Women who died before labor were excluded from analyses because these women usually sought care only after developing severe complications at home. So, it was often too late to prevent death by the time these women were admitted into the referral hospital.

### Data collection

Data was collected from medical records by trained midwives who were supervised by the national coordinators of the survey. In each country, data was collected on a daily basis on every woman who gave birth in every selected facility. The database for this study included information on maternal demographic characteristics, obstetric history (gravidity, parity and previous CS), prenatal care (number of antenatal care visits during the current pregnancy), management of labor and delivery, obstetric complications, and the vital status of both mother and child until hospital discharge (Table S1). To avoid under- reporting of hospital-based maternal mortality, a complementary procedure was carried out to identify the eligible maternal deaths among all the female deaths that occurred in the facility using the various registries available (admissions, hospitalizations, operating theatres and morgues).

For the variables related to the diagnosis of clinical signs or symptoms related to obstetric complications, only these which were collected during the first 24 hours after admission were taken into account for the analyses. This specific set of variables was chosen for clinical reasons and was supported by previous work in low-resources countries [17], [18], [19]. We assume that all these potential predictive clinical signs or symptoms should be easily measured by health care staff with various competences.

Quality control was performed on site by the study coordinator, who selected a random sample of 5% of all patient forms in each site, and compared the following data with information available in the clinical records: age, parity, diagnosed pathology or complication, mode of delivery and vital status of the mother at discharge. An agreement rate of 80% or higher between the two sources of information was considered acceptable. If the rate of agreement was between 50% and 80%, the coordinator repeated the procedure with another 10% random sample of forms. If the rate of agreement was less than 50%, he controlled all the forms in a given site. At the end of each trimester, the electronic record containing all relevant clinical data was transmitted to the trial's coordinating center at the University of Montreal for quality control (missing data and inconsistent/implausible values).

### Statistical analysis

In-hospital maternal mortality was the outcome of interest. Accordingly, all deaths that occurred during the hospital stay (before hospital discharge) among women who had delivered in the selected centers during the study period were counted as the outcomes. In this study, because of the very low prevalence of the outcome (maternal in-hospital death) in the large data base of the study resulting in highly unbalanced distribution of the dependent variable, standard prediction methods based on logistic regression or classification trees are not appropriate [20], [21]. The method extracts specific multivariate ‘risk patterns’, which identify subgroups of patients for whom the prevalence of the outcome (here: risk of maternal death) is higher than in the entire study population. We describe the method elsewhere [22]. Briefly, the first step on the analysis is a training step which use the Apriori algorithm developed by Agrawal and Coll [23], one of the most widely used algorithms in the data mining literature, to discover frequent ‘risk patterns’. Since the Apriori algorithm results in a very large number of ‘risk patterns’, the second step is a pruning step that aims to produce a smaller set of non redundant ‘risk patterns’. In the third step, we validate and combine the ‘non redundant risk patterns’ in a classification rule. In the last step, we assess the performance of the classification rule by computing its sensibility and specificity (with 95% confidence interval). This process requires to split the database randomly into three subsets, each comprising 33% of all the patients. The first and second steps are run using the ‘training’ dataset. The first step results in 929 frequent rules and the second step results in 575 no redundant rules. The third step is run using the ‘validation’ dataset. After this validation step the number of remaining rules is 20. The fourth step is run using the ‘test’ dataset.

Descriptive analyses were carried out with Epi-Info software (version 3.5.3). All analyses related to the proposed classification method were performed using the packages “arules” in the version 2.15.1 (2012-06-22) of the programming environment R [24].

## Results

Among the 89,847 enrolled patients, 329 were excluded (175 women who died before delivery and 154 women transferred to another health facility), leaving 89,518 women for the analyses, 29,840 for the ‘training’ dataset, 29,839 for the ‘validation’ data set and 29,839 for the ‘test’ dataset. Table 1 presents in-hospital maternal mortality rates, according to the type of institution and the country, and some indicators of the risk of the pregnant population served by each type of institution. The proportions of high risk women were higher in hospitals outside the capital that in hospitals located in Dakar or Bamako. In each category of health care facility, the mean length of hospital stay was higher for women with a cesarean section than for women who deliver by vaginal way. Hospital-based mortality rates in district hospitals, regional hospitals and in hospitals in the capitals (Dakar or Bamako) varied between centers, ranging from 0.14% to 3.66% (Median = 1.07%), 0.81% to 3.26% (Median = 1.60%), and 0.06% to 2.01% (Median = 0.26%), respectively. Hemorrhage, pre-eclampsia/eclampasia and indirect causes (anemia, malaria and chronic disease) were the major causes of maternal mortality.

**Table 1:** Number of patients, in-hospital maternal mortality rate, age, type of admission, complication and caesarean delivery rates and length of stay, by type of hospital and country.

Country and type of hospital	Patients N	Mortality° %	Age in years, Mean (SD)	Women referred§ %	Women with complication* %	Caesarean deliveries %	Length of hospital stay in days,mean (SD)	
							Vaginal delivery	Cesarean delivery
**Senegal**								
Hospitals in Dakar (capital city)	15606	0.29	27.9 (6.7)	15.6	18.0	19.5	1.1 (2.5)	5.8 (3.6)
Regional hospitals outside of Dakar	22617	1.20	26.2 (6.7)	37.8	34.7	23.7	1.4 (3.0)	6.2 (4.3)
District hospitals outside of Dakar	8629	0.84	24.9 (6.7)	22.4	22.4	7.6	1.4 (2.5)	7.0 (4.0)
**Mali**								
Hospitals in Bamako (capital city)	24118	0.1	25.0 (6.5)	16.8	13.7	16.6	0.3 (1.2)	3.5 (2.6)
Regional hospitals outside of Bamako	7678	0.79	25.2 (6.7)	21.7	28.2	20.0	0.6 (3.6)	4.8 (2.7)
District hospitals outside of Bamako	10870	1.10	25.9 (7.1)	29.3	27.1	21.1	1.2 (2.2)	7.0 (5.0)
**Total**	89518	0.69	25.9 (6.8)	24.4	23.5	18.9	1.0 (2.5)	5.5 (4.0)

° Proportion of maternal deaths before hospital discharge among women giving birth in the facility during the study period.

§ Referral from another hospital.

* One of the following obstetric complications: ante- or post-partum hemorrhage, pre-eclampsia/eclampsia, prolonged obstructed labor,

Uterine rupture, puerperal sepsis.

Since the class of interest has low frequency of occurrence, it is preferable to consider the local support instead of the support [25], [26], [27]. The support of a risk pattern is the probability that a patient belongs to that risk pattern and dies; the local support of a risk pattern is the probability that a patient dies given that the risk pattern holds. We can deduce the support of a rule from its local support. Thus, in our procedure of generation of all the candidate rules we set the training parameters as follows: minimum local support equal to: 9%, 10%, 15%; minimum confidence equal to: 2.1%, 2.8%, 3.5%; and maximum length equal to: 3, 4. Combining these different values of training parameters (local support, confidence, length) has as a result, eighteen (18) sets of representative rules of which each one give a classifier. These classifiers are used to build a ROC curve. This ROC curve is used to select the optimal classifier according to sensitivity and specificity and misclassification rate if necessary. Table 2 presents the selected classification rule build with twenty non redundant ‘risk patterns’. The twenty ‘risk patterns’ composing our optimal classifier, are defined by different combinations of the following fourteen variables: age, parity, previous cesarean section, five variables related to current pregnancy (prenatal care attendance, vaginal bleeding during pregnancy, Malaria, premature rupture of the membranes and multiple pregnancy), and six variables related to the labor or delivery (referral from another health facility, labor induction, mode of delivery (emergency ante-partum cesarean delivery and intra-partum cesarean delivery), hemorrhage, Prolonged/obstructed labor, and uterine rupture). Table 3 presents the operational definitions of these fourteen variables.

**Table 2:** Classification association rule predicting in-hospital maternal mortality* among patients delivering in 46 hospital in Senegal and Mali.

Risk patterns	Prevalence of the pattern among all patients, %	Prevalence of the pattern among women who died, %	Relative risk [95% CI]
1. Patient with hemorrhage, No vaginal bleeding during pregnancy and referred from another health facility	1.78	35.87	30.7 [23.6–39.81]
2. Patient with hemorrhage, No vaginal bleeding during pregnancy and Parity equal to 5 and more	0.98	21.97	28.2 [20.9–37.8]
3. Patient Referral from another health facility with Uterine rupture and Prolonged/obstructed labor	0.42	10.31	27.11 [18.2–40.2]
4. Patient with hemorrhage, No vaginal bleeding during pregnancy and Age equal to 35 years and more	0.44	10.76	26.93 [18.2–39.6]
5. Patient Referral from another health facility with Uterine rupture and No Previous caesarean section	0.43	10.31	26.28 [17.6–39.0]
6. Patient with hemorrhage, No vaginal bleeding during pregnancy and no malaria diagnosed	2.99	44.39	25.84 [20.0–33.3]
7. Patient with hemorrhage, referred from another health facility and Parity equal to 5 and more	1.41	20.17	17.66 [12.9–24.1]
8. Patient with hemorrhage, referred from another health facility and no labor induction	3.37	36.32	16.33 [12.5–21.3]
9. Patient with hemorrhage, referred from another health facility and no malaria diagnosed	3.88	39.46	16.14 [12.4–20.9]
10. Patient with hemorrhage, Antenatal care attendance between 1–3 and Prolonged/obstructed labor	1.01	13.45	15.10 [10.4–21.8]
11 Patient with hemorrhage, Parity equal to 5 and more and No Labor induction	1.75	21.07	14.911 [10.9–20.3]
12. Patient Referral from another health facility with No Vaginal bleeding during pregnancy and emergency ante-partum cesarean delivery	1.14	11.21	10.85 [7.2–16.2]
13. Patient with no Vaginal bleeding during pregnancy, No Premature rupture of the membranes and emergency ante-partum cesarean delivery	1.98	17.04	10.13 [7.2–14.2]
14. Patient referred from another health facility with Intra-partum cesarean delivery and Parity equal to 5 and more	1.51	11.21	8.22 [5.4–12.3]
15. Patient referred from another health facility with no Vaginal bleeding during pregnancy and Parity equal to 5 and more	4.03	25.11	7.96 [5.9–10.7]
16. Patient referred from another health facility with no Vaginal bleeding during pregnancy and age equal to 35 years and more	2.20	14.34	7.43 [5.1–10.7]
17. Patient referred from another health facility with Intra-partum cesarean delivery and Antenatal care attendance equal to 1,2 or 3	4.09	21.07	6.25 [4.5–8.5]
18. Patient referred from another health facility with No Vaginal bleeding during pregnancy and emergency ante-partum cesarean delivery	7.39	31.83	5.84 [4.4–7.7]
19. Patient referred from another health facility with No Antenatal visits and No Multiple pregnancy	3.59	15.69	4.99 [3.4–7.1]
20. Patient referred from another health facility with Antenatal care attendance equal to 1,2 or 3 and Prolonged/obstructed labor	5.26	21.52	4.93 [3.6–6.7]

* The set of the twenty risk patterns predicts in-hospital maternal mortality with 81.41% sensitivity (95% CI = [77.12%–87.70%]) and 81.6%specificity (95% CI = [81.16%–82.02%]).

**Table 3:** Definitions of the variables included in the association classification rules.

Variable	Definition
Age	Use the patient's age (year) at last birthday
Parity	number of full-term pregnancy of the patient
Previous caesarean section	Patients who had to give birth by caesarean section before the current pregnancy
Antenatal care attendance	Use the number of antenatal visits during the current pregnancy
Malaria	Patient who had diagnosed malaria in the current pregnancy
Multiple pregnancy	Patient who gave birth more than one newborn.
Vaginal bleeding during pregnancy	Woman who had diagnosed vaginal bleeding in the current pregnancy
Premature rupture of the membranes	Woman who had diagnosed premature rupture of the membranes in the current pregnancy
Ante- or post-partum hemorrhage	Vaginal bleeding before delivery (ante-partum) or excessive blood lost during the third stage of labor (immediate post-partum)
Referral from another health facility	Patient who was admitted in another health care facility and secondarily referred to the hospital, whatever the transportation mean.
Labor induction	Induced labor before delivery
Intra-partum cesarean delivery	Caesarean delivery during the labor.
Emergency ante-partum caesarean delivery	Caesarean delivery before the onset of labor with no enough time to schedule the surgery
Uterine rupture	The occurrence of clinical symptoms (pain, fetal distress, acute loss of contractions, hemorrhage) or intrauterine fetal death that led to laparotomy, at which the diagnosis of uterine rupture is confirmed; or laparotomy for uterine rupture after vaginal birth.
Prolonged/obstructed labor	Prolonged/obstructed labor before delivery

The highest relative risks (RR≥28) are related to the following patterns: (i) “Patients with hemorrhage, no vaginal bleeding during pregnancy and referred from another health facility” and (ii) “Patients with hemorrhage, no vaginal bleeding during pregnancy and Parity equal to 5 and more ” (last column of Table 2). The highest ‘risk pattern’ prevalence among women who died before hospital discharge are related to the following ‘risk patterns’: (i) “Patient with hemorrhage, no vaginal bleeding during pregnancy and no malaria diagnosed”; and (ii) “Patient with hemorrhage, Referred from other health facility and No malaria diagnosed”. Hemorrhage and referral from another health facility are the conditions which are included in nineteen of the twenty ‘risk patters’ shown in Table 2. The resulting classification rule predicts in-hospital maternal mortality with 82.41% sensitivity (95% CI = [77.127%–87.70%]) and 81.6% specificity (95% CI = [81.16%–82.02%]) (see table 4).

**Table 4:** Sensibility and specificity (and 95% CI) associated with the classification association rule among the patients selected in the ‘test’ dataset (N = 29 838).

		Observed mortality	
		Death	No Death
Predicted mortality	Death	164	5454
	No Death	35	24186
Total		199	29640
		Se = 82.41% 95% CI = 77.12%–87.70%	Sp = 81.6% 95% CI = 81.16%–82.02%

**Figure 1:**
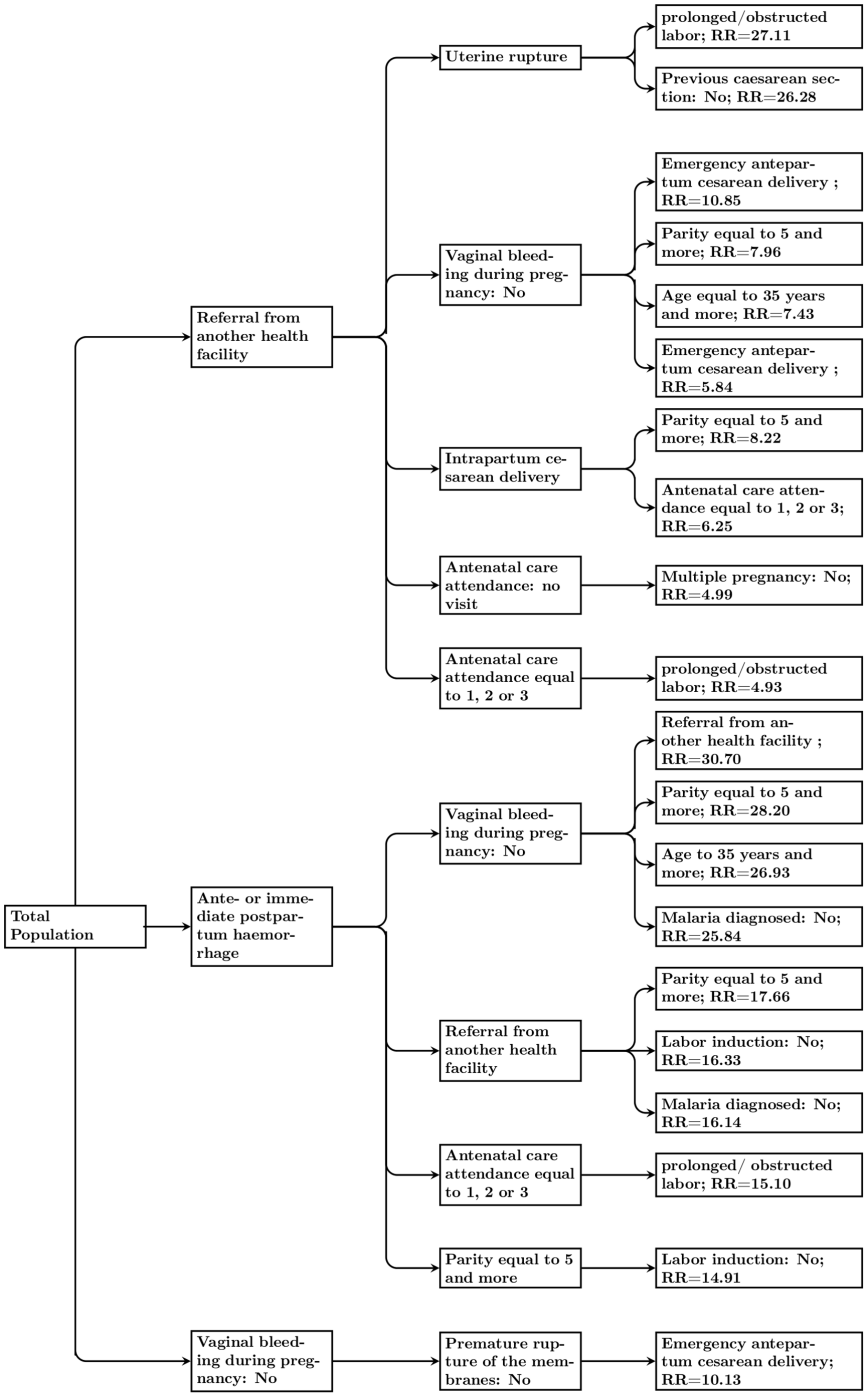
Tree representation of the twenty rules mined.

For clinical use, the resulting tree structure, shown in Figure 1, can be used to visualize the rules mined. Each branch of the tree constitutes a risk pattern, which relative risk of in-hospital mortality is given at the terminal node of the branch. And for each node, a variable value pair is represented. Immediately below each branch, we show the next split of the sub-population classified to a given branch which is based on a set of new value pair. For example, according to Figure 1, “patients who have hemorrhage and no vaginal bleeding during pregnancy and are referred from another health facility” are 30.70 times more likely to die than the population average. For the patients who have “hemorrhage, no vaginal bleeding during pregnancy and parity equal to 5 and more ”, the relative risk (RR) of in-hospital-death rises to 28.20 and for the patients who are referred from another health facility and have an uterine rupture and a prolonged/obstructed labor, the RR rises to 27.11. Similar interpretation of the tree structure can be made for each main node of the tree that identify, respectively, patients with no antenatal care attendance, patients with emergency ante-partum cesarean delivery or referred from other health facilities.

## Discussion

The rules identified by our association rule method provide useful information to health care professionals, and can serve as clinical algorithms for the triage of high-risk patients who are admitted in referral hospitals in Mali and Senegal.

Hospital-based maternal mortality rates varied considerably between centers, ranging from 0.03% to 3.66% of all deliveries. As reported elsewhere in SSA countries, hemorrhages including uterine rupture and hypertensive complications are the major causes of maternal mortality [4], [5], [6], [7], [8], [9], [10], [11]. As expected, the clinical signs or symptoms related to these complications were associated with hospital-based maternal mortality: [3], [4], [5], [6], [7], [8], [9], [10], [11]. Furthermore, the classification approach developed in this study shows that patients who were referred from other health care facilities or had emergency ante-partum cesarean delivery (ante- and intra-partum) also have an increased risk of hospital-based mortality. These results are in accordance with other studies in Latin America, Asia and Africa [7], [8], [9], [10], [17], [18], [19].

This study is one of the largest one on hospital-based maternal mortality in Sub-Saharan Africa. The participant hospitals were representative of the existing health system in both countries, taking into account the full variety of contexts and levels of care. The large database and the system of data control we implemented in all participating hospitals allowed us to obtain reliable information on maternal outcomes [16]. Our findings may be generalized to other countries with similar health care systems and other referral hospitals with similar population.

Potential limitations of this study should be taken into account in interpreting our results. First, data are restricted to in-hospital critical events; therefore maternal mortality and late complications as puerperal infection that occurred after hospital discharge may be underestimated. Second, diagnoses during current pregnancy (e.g., pre-eclampsia) may have been reported differently among the participating hospitals. These potential misclassifications lead to categorizing some ‘exposed’ women (e.g. those with pre-eclampsia) as ‘no exposed’ and might have modified the interactions between the variables. Third, our survey includes only hospitals that have a functional operating room offering comprehensive emergency obstetric care. The results, therefore, cannot be generalized to primary health care facilities or community health centers that offer basic emergency obstetric care. For the same reasons, maternal mortality in the population or in community centers cannot be inferred.

The classification rule established in this study confirms that patients with uterine rupture, hemorrhage, prolonged/obstructed labor or parity equal to 5 and more, should be managed with high priority by qualified health professionals in comprehensive EmOC services [2], especially if the patient is referred from another health facility. Given the shortage of qualified health care professionals (midwives and doctors) in Mali and Senegal, many tasks are shared with less qualified health personnel (students, matrons, nurse-assistants). This personnel may play a crucial role in improving maternal outcomes in referral hospitals if they are involved in the triage of high risk patients. Specifically, our results indicate that they should be trained to detect uterine rupture and hemorrhage. The required tasks and actions are quite specific and simple: ask about pain and contractions, as well as vaginal bleeding during pregnancy, measure blood pressure, protein dipstick, detect excessive blood loss and seizures. Even non-qualified health personnel could detect, at admission or during labor/delivery, the following alarm signs: acute pain and loss of contractions, blood pressure >140/90 mmHg, proteinuria>1+, hemorrhage; and they should then immediately alert qualified professionals if any of these signs are detected.

The early detection of these signs of complication, and immediate management by midwives or doctors would improve maternal outcomes [2], [4], [9]. Moreover, ensuring a prompt access to the emergency obstetric care for women with complications requires that health centers are equipped to deal with the emergencies they are facing, and that timely care in referral hospitals, if needed, is not hindered by delays in transportation. A maternity referral system in Mali, that attempts to remove geographic and financial barriers, that ensured basic and comprehensive emergency obstetric care, transportation to obstetric health services and community cost-sharing schemes, has produced a substantial reduction in maternal mortality rates [10]. Other mechanisms to improve referral and transportation of mothers with complications have also to be explored in low-resources settings in order to improve maternal and prenatal outcomes.

## Conclusion

An important advantage of the association rules established in this study might be a timely identification by qualified or non-qualified health professionals of mothers at highest risk of in-hospital mortality, who can be then offered high priority emergency obstetric care. Such strategy should target all pregnant women attending referral hospitals in Senegal and Mali and aim to detect and manage life-threatening complications via evidence-based interventions, with intensive monitoring of the women who have emergency ante-partum cesarean delivery. Additional studies, in other settings, are necessary to assess the impact of this strategy on the reduction of the time-to-care, case-fatality rates and overall in-hospital maternal mortality. This strategy will offer even more benefits if combined with interventions improving maternal referral system.

## Supporting Information

Table S1 **List of variables used for predicting in-hospital maternal mortality (Doc).**
(DOCX)
