# Supplementary material for: Predicting In-Hospital Maternal Mortality in Senegal and Mali
Source: PLoS One. 2013 May 30;8(5):e64157. doi: 10.1371/journal.pone.0064157 (PMC3667861; doi:10.1371/journal.pone.0064157)
Supplement: Table S1 — List of variables used for predicting in-hospital maternal mortality (Doc). (DOCX) [file pone.0064157.s001.docx]

| Variable | Code Values |
| --- | --- |
| *Previous medical history* |  |
| Age group (16y and less; 17-34; 35y and more) | 1, 0, 2 |
| Parity (nulliparous, 1-4, 5 and more) | 1, 0, 2 |
| Chronic arterial hypertension | 0,1 |
| Chronic Cardiac/renal disease | 0,1 |
| Chronic pulmonary disease | 0,1 |
| Sickle Cell disease | 0,1 |
| Previous caesarean section | 0,1 |
| *Current pregnancy* |  |
| Gestational hypertension | 0,1 |
| Pre-eclampsia/eclampsia | 0,1 |
| Vaginal bleeding during pregnancy (near the term) | 0,1 |
| Severe chronic anemia | 0,1 |
| Gestational diabetes | 0,1 |
| Premature rupture of the membranes | 0,1 |
| Urine tract infection/pyelonephritis | 0,1 |
| HIV/AD | 0,1 |
| Malaria | 0,1 |
| Multiple pregnancy | 0,1 |
| Antenatal care attendance (no visit, 1-3, 4 and more) | 2, 1, 0 |
| *Labor and delivery* |  |
| Referral from another health facility | 0,1 |
| Labor induction | 0,1 |
| Mode of delivery |  |
| normal vaginal | 0 |
| forceps/vacuum | 1 |
| emergency ante-partum cesarean delivery | 2 |
| Intra-partum cesarean delivery | 3 |
| elective cesarean delivery | 4 |
| Ante- or immediate postpartum hemorrhage | 0,1 |
| Prolonged/obstructed labor | 0,1 |
| Uterine rupture | 0,1 |

List of the twenty four variables used for predicting in-hospital maternal mortality
